# T-cell immunity in the experimental autoimmune vasculitis rat model

**DOI:** 10.1016/j.jtauto.2025.100305

**Published:** 2025-08-07

**Authors:** Ye Zeng, Erika Boschmann, Julia Kotte, Ming Sun, Lennart Hess, Sebastian Dolff, Tanja Hinkeldein, Janna Hagedorn, Robert Langer, Pieter van Paassen, Jan Damoiseaux, Jan Willem Cohen Tervaert, Oliver Witzke, Andreas Kribben, Benjamin Wilde

**Affiliations:** a Department of Nephrology, University Hospital Essen, University of Duisburg-Essen, Essen, Germany; b Department of Infectious Diseases, University Hospital Essen, University of Duisburg-Essen, Essen, Germany; c Department of Internal Medicine, Section of Immunology, Maastricht University Medical Center, Maastricht, the Netherlands; d Central Diagnostic Laboratory, Maastricht University Medical Center, Maastricht, the Netherlands; e Department of Medicine, Division of Rheumatology, University of Alberta, Edmonton, Canada; f School for Mental Health and Neurosciences (MHeNS), Maastricht University, Maastricht, the Netherlands

**Keywords:** ANCA vasculitis, Th17 cells, Th1 cells, MPO, PR3

## Abstract

ANCA-vasculitis (AAV) is a small-vessel vasculitis characterized by the presence of autoantibodies against proteinase-3 (PR3) or myeloperoxidase (MPO). The dynamics of the T-cell response within tissues is studied best in animal models. It was the aim to analyze the lesional T-cell dynamics in the experimental autoimmune vasculitis model.

Female Wistar Kyoto-rats were immunized with human MPO emulsified in complete Freund's adjuvant. Control animals received complete Freund's adjuvant without MPO. Selected groups received anti-IL17A treatment. Lesional T-cells from kidneys were assessed by flow cytometry (FACS), realtime polymerase chain reaction (PCR) and EliSpot.

All animals immunized with MPO developed signs of vasculitis. At week six, lung damage expressed as petechial bleeding score and renal damage quantified by albuminuria were highest. As analyzed by FACS, the fraction of renal Th17 cells peaked at week six in MPO rats equaling the proportion of Th1 cells. MPO-specific renal Th1 and Th17 cells were detectable by EliSpot at weeks four and six post-immunization in MPO-immunized rats being absent in control rats. Neutralization of IL-17A did not affect the development of humoral and cellular anti-MPO immunity. Likewise, pulmonary and renal vasculitis were not ameliorated.

In summary, the dynamics of the lesional T-cell response in the EAV model shows a major participation of MPO-specific Th17 and Th1 cells in renal vasculitis. Simple cytokine neutralization was not efficacious in this disease model so that combined neutralization approaches should be studied further.

## 1 Introduction

ANCA-vasculitis (AAV) is a small-vessel vasculitis which is characterized by the presence of autoantibodies against proteinase-3 (PR3) or myeloperoxidase (MPO) [1,2]. Next to the humoral immune system, the cellular immunity has a major role in disease mechanisms. Persistent activation of T-cells in patients with AAV has been reported by several groups; disturbed immune tolerance, aberrant co-stimulation and co-inhibition are suggested to contribute to these T-cell abnormalities [[3], [4], [5]]. Circulating T-cells are polarized towards Th17 in AAV [[3], [4], [5]]. There is a lack of information on the inflammatory processes on tissue level; the inflamed tissues are not easily accessible in patients and thus, animal models are important to decipher the lesional inflammatory processes within the tissues. The experimental autoimmune vasculitis rat model was originally developed by Little et al. [6]. The advantage over other available models is that 1. disease induction is based on formation of antigen-specific humoral and cellular immunity 2. systemic vasculitis with pulmonary and renal manifestations occurs after disease induction and 3. the disease severity is not overwhelming so that therapeutic approaches can be evaluated. The dynamics of the lesional T-cell response in the kidney has not been studied so far in this model. Thus, deciphering the timing and type of renal T-cell inflammation may provide important insights for further use of this model.

## 2 Material and methods

### 2.1 Experimental autoimmune vasculitis model

The model was adopted from Little et al. [6]. Female WistarKyoto rats weighing 120–160 g were immunized with subcutaneous injection of human myeloperoxidase (MPO, 1600 μg/kg body weight, Merck Chemicals, Nottingham, UK) emulsified in complete Freund's adjuvant (Sigma Aldrich, Taufkirchen, Germany). On the day of immunization and on day two, the rats received an intraperitoneal injection of pertussis toxin (800 ng) to booster the immunization against MPO. Control rats were treated the same way but received Freund's adjuvant without MPO. Two, four and six weeks after immunization, rats were culled under deep anesthesia and organs were harvested for further analysis. For the purpose of IL-17A neutralization, a monoclonal rat IgG2a anti-mouse/rat IL-17A (clone eBio17CK15A5, Thermo Fisher Scientific, Darmstadt, Germany) was used and injected intraperitoneally twice per week at a fixed dose of 100 μg per animal. Selected MPO-immunized animals were treated for either six weeks starting one day before immunization with MPO or three weeks starting on day 21 after immunization with MPO. Control animals received a corresponding isotype control at the same dosage (Rat IgG2a κ, Biolegend, Amsterdam, The Netherlands). For histology, parts of the kidneys were fixed in formaline and embedded in paraffine. Periodic acid Schiff staining of kidney sections was done and slides were judged by two independent observers. 100 glomeruli were counted per slide and the amount of abnormal glomeruli was noted. If signs of necrotizing glomerulonephritis were present such as loop necrosis, extracapillary proliferation or rupture of Bowman's capsule, a glomeruli was judged as abnormal. To determine the degree of albuminuria, rats were placed in metabolic cages and urine was collected over 12 h. Albuminuria was determined using a commercially available ELISA kit (anti-rat albumin ELISA kit, Alpco, Salem, USA). Serum samples were obtained by drawing whole blood from the tail vein. The petechial bleeding score was calculated to determine the extent of pulmonary vasculitis. The lung was harvested and the visible petechial lesions on the lung surface were counted. Below 100 visible lesions, the count was given as the exact number of lesions. If more than 100 distinct lesions visible, the count was set to 100. If more than 200 distinct lesions were counted, the count was set to 200. The animal study was approved by the local authorities (LANUV NRW, Germany, G1351/13 and G1692/18).

### 2.2 Determination of antibodies against myeloperoxidase

96-well microtiter plates (Greiner Bio one, Frickenhausen, Germany) were coated with human myeloperoxidase (Merck Chemicals) diluted in coating buffer (endconcentration 2ug/ml) over night at 4 °C. After washing with PBS containing Tween20 and blocking with PBS containing 1 % BSA, serial dilutions of serum samples were assayed and incubated for 1 h at 37 °C. Secondary antibody (anti-rat IgG H + L, HRP conjugated, Jackson Immuno, Cambridgeshire, UK) was added at a dilution of 1:20.000 after rinsing and washing followed by an incubation period of 1 h at 37 °C. TMB solution was added after rinsing and washing. Stop solution containing HCl was applied after 5 min and the absorption was measured within 30 min on an ELISA plate reader (Mithras LB 940, Berthold Technologies, Bad Wildbad, Germany). In addition to ELISA, selected serum samples from rats were tested in an immune-fluorescence assay. For this purpose, rat neutrophils were isolated from whole blood by density gradient centrifugation. Rat neutrophils were transferred to glass slides and fixed with cold ethanol at −20 °C for 20 min. Subsequently, the slides were air dried. The slides were then incubated with diluted serum samples for 30 min at room temperature followed by PBS rinsing. A secondary anti-Rat IgG FITC-coupled antibody (Jackson Immuno) was added for 20 min at room temperature followed by thorough rinsing in PBS. DAPI containing mounting medium was used to coverslip the slides (Vector Laboratories, Newark, CA).

### 2.3 Preparation of tissues for cell isolation

Mononuclear cells were isolated from spleen and from renal tissue. Spleen and renal tissue were cut, minced and the tissue was filtered through a 50 μm cell strainer to obtain a single cell suspension. Red blood cell lysis of spleen cells was done with ammonium chloride. Spleen cells were used after this step for further cell culture procedures. Mononuclear cells from renal tissue were further purified by magnetic isolation. The single cell suspension from renal tissue was incubated with anti-rat CD45 (PE labelled, Miltenyi Biotec, Mönchengladbach, Germany) for 30 min at 4 °C followed by two washing steps with PBS containing 0.5 % BSA and 2 mM EDTA. Then, the cell suspension was incubated with anti-PE coupled to microbeads (Miltenyi Biotec) for 15 min at 4 °C. LS columns and Quadro MACS separator (both Miltenyi Biotec) were used to obtain the magnetically labelled leukocytes.

### 2.4 Flow cytometry

Isolated mononuclear cells were stimulated with PMA (Sigma Aldrich, 50 ng/ml) and Ionomycin (Sigma Aldrich, 1 μg/ml) for 16 h at 37 °C, 5 % CO_2_ in RPMI 1640 Glutamax (Thermo Fisher Scientific) supplemented with non-essential amino acids, 10 % fetal calf serum (Biowest, Nuaillé, France) and 2 % penicillin/streptomycin (Thermo Fisher Scientific). For the last 4 h of incubation, Brefeldin A (Sigma Aldrich, 5 μg/ml) was added. Unstimulated cells were used as controls. Following stimulation, cells were labelled with anti-rat CD45 (either FITC or PE, Thermo Fisher Scientific) and anti-rat CD3 (APC, Thermo Fisher Scientific). Afterwards, cells were fixed and permeabilized (Cytofix/Cytoperm, BD Biosciences, Heidelberg, Germany) to stain for IL-17A (anti-rat IL-17A PE, Thermo Fisher Scientific) and IFNγ(anti-rat IFNγFITC, Thermo Fisher Scientific) followed by washing steps. The samples were measured immediately on the flow cytometer (FACS Navios, Beckman Coulter).

### 2.5 Elispot

Elispot was used to detect MPO-specific T-cells. Briefly, cells were rested overnight in RPMI 1640 Glutamax (Thermo Fisher Scientific) supplemented with non-essential amino acids, 10 % fetal calf serum (Biowest, Nuaillé, France) and 2 % penicillin/streptomycin (Thermo Fisher Scientific). The cells were cultured with human MPO (15 μg/ml, Merck Chemicals) on elispot plates either coated with anti-rat IFNγ (rat IFN-y ELISpot Plus, HRP, Mabtech, Nacka Strand, Sweden) or anti-rat IL-17A (mouse/Rat IL-17A Elispot Ready-Set-Go, HRP, Ebioscience) capture antibodies for 16 h. Cells cultured without MPO served as negative controls whereas cells stimulated with PMA (5 ng/ml) and Ionomycin (2ug/ml) served as positive controls. The kits were used according to the instructions by the manufacturer. Dots were counted on an Elispot Reader (Classic, AID, Straβberg, Germany).

### 2.6 Realtime polymerase chain reaction

RNA was isolated from tissues preserved in RNAlater using a commercially available kit (RNAeasy mini kit, Qiagen, Hilden, Germany). Purity was checked with a spectrophotometer (Nanodrop). cDNA was transcribed with the Quantitect reverse transcription kit (Qiagen). Pre-designed taqman primers for the rat targets β-actin, CXCL1, CCL20, IL-17A, IFNγ and IL-10 were used (Thermo Fisher Scientific). β-actin served as housekeeping gene. The experiments were performed on a Step One plus Realtime PCR system and the data was analyzed with the StepOne Software Version 2.3 (both Thermo Fischer Scientific). The ΔΔCT method was applied for analysis.

### 2.7 Cell culture

NRK52E cells (Leibniz Institute, DSMZ, Braunschweig, Germany) were cultured in complete DMEM supplemented with 5 % fetal calf serum (Biowest) and 1 % penicillin/streptomycin. (Thermo Fisher Scientific). The cells were either stimulated with rat recombinant IL-17A (100 ng/ml, R&D Systems), rat recombinant TNFα (100 ng/ml, Sigma Aldrich) or a combination of both in presence or absence of the anti-IL-17A antibody (50 μg/ml, clone eBio17CK15A5, Thermo Fisher Scientific). Unstimulated cells served as controls.

### 2.8 Statistics

For the unpaired comparison of two groups, the non-parametric Mann-Whitney *U* Test was applied. To compare more than two unpaired groups, one-way ANOVA was performed with Tukey's multiple comparisons test for statistical analysis. Correlation analysis was performed with spearman's rank correlation. A p-value below 0.05 was considered significant.

## 3 Results

### 3.1 Immunization with myeloperoxidase induces MPO-specific immunity and vasculitis

**Fig. 1:**
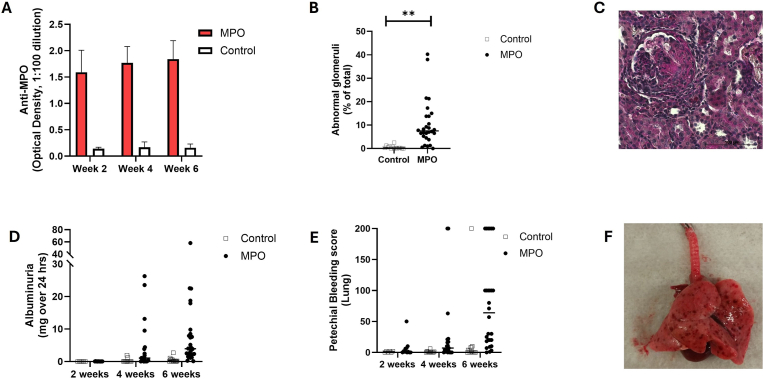
Characteristics of the experimental autoimmune vasculitis model. **(A)** Antibodies against MPO are detectable from week two onward and persist throughout week six in MPO-immunized animals. The data is depicted as mean with SD (week two: MPO n = 51, control n = 32; week four: MPO n = 39, control n = 27; week six: MPO n = 28, control n = 15). Levels were determined by ELISA and samples were diluted 1:100. The optical density is depicted. **(B)** MPO-immunized animals developed renal vasculitis and the degree of renal damage was quantified by histology (week six, MPO: n = 28, control n = 14). Two independent observers judged 100 glomeruli and counted the number of abnormal glomeruli showing extracapillary proliferation, necrosis or rupture of bowman's capsule. The horizontal line depicts the median. **(C)** Representative PAS stained section of an MPO-immunized animal culled at week six. The section depicts an abnormal glomeruli showing extracapillary proliferation. Magnification is 400x. **(D)** Significant albuminuria is only detected in MPO-immunized animals and peaks at week six after immunization (MPO: n = 28, control n = 15). The horizontal line depicts the median. **(E)** MPO-immunized animals develop pulmonary vasculitis. In case of animals with pulmonary vasculitis, petechial bleeding spots were counted on the surface of the lung (week two: MPO n = 12, control n = 6; week four: MPO n = 19, control n = 12; week six: MPO n = 28, control n = 15). The spots were counted per animal and the number of spots represented the petechial bleeding score. The horizontal line depicts the median. **(F)** Explanted lung of an MPO-immunized animal. Petechial bleedings are present on the surface of the lung. Mann-Whitney *U* Test was used to compare two different groups and a p-value below 0.05 was considered significant. ∗∗p-value <0.005.

Two weeks after immunization with human MPO, antibodies against MPO were detectable. These antibodies were reactive with ethanol fixed rat neutrophils and a peri-nuclear staining pattern was observed suggesting the reactivity of anti-human MPO antibodies with rat myeloperoxidase. This was also reported previously by Foucher et al. and by Little et al. [6,7]. Antibody titers against MPO were highest at week six post immunization (Fig. 1A). Control animals which received Freunds adjuvant only did not develop antibodies against human MPO (Fig. 1A). All animals immunized with MPO developed signs of vasculitis. At week six, lung damage expressed as petechial bleeding score and renal damage quantified by albuminuria were highest (Fig. 1B–F). Splenic Th1 and Th17 cells were analyzed by FACS. The fraction of splenic Th1 and Th17 cells was comparable between MPO and control animals at all timepoints (Table 1). Interestingly, MPO-specific splenic Th17 cells were detectable in all MPO animals at week six as measured by EliSpot. In contrast, MPO-specific splenic Th1 cells were detectable only in a minority of MPO immunized animals (data not shown).

**Table 1:** Proportion of splenic Th1 and Th17 cells over time as measured by flow cytometry. Statistical differences were calculated by using non-parametric Mann-Whitney *U* Test.

	Group	Timepoint	% of splenic CD3^+^ T-cells (mean ± SD)	p-value vs. control
**Splenic Th17 cells**	MPO, n = 6	Week Two	3.07 ± 1.6	p = 0.33
	Control, n = 6	Week Two	2.15 ± 0.6	
	MPO, n = 4	Week Four	1.89 ± 0.4	p = 0.35
	Control, n = 6	Week Four	1.51 ± 0.6	
	MPO, n = 12	Week Six	1.82 ± 0.6	p = 0.46
	Control, n = 11	Week Six	1.56 ± 0.5	
**Splenic Th1 cells**	MPO, n = 6	Week Two	2.41 ± 0.9	p = 0.70
	Control, n = 6	Week Two	2.04 ± 0.5	
	MPO, n = 4	Week Four	2.34 ± 1.3	p = 0.48
	Control, n = 6	Week Four	1.61 ± 0.6	
	MPO, n = 12	Week Six	3.16 ± 1.4	p = 0.24
	Control, n = 11	Week Six	2.49 ± 0.7	

### 3.2 Dynamics of renal inflammation

**Fig. 2:**
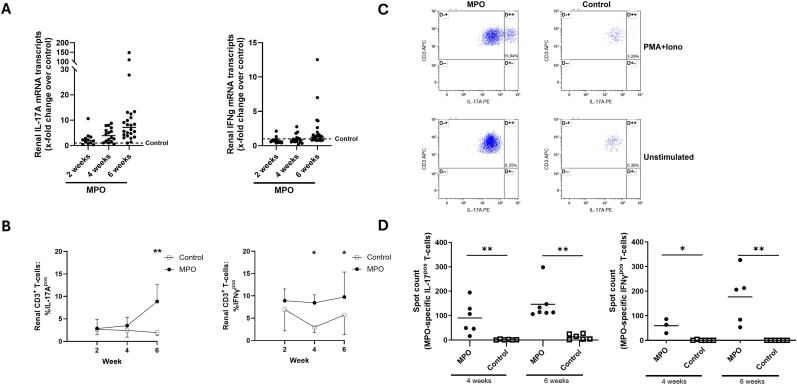
Dynamics of T-cell immunity in the experimental autoimmune vasculitis model. **(A)** mRNA analysis of the kidney by RT-PCR revealed a persistent rise of IL-17A transcripts in MPO-immunized animals over six weeks. In contrast, IFNγtranscripts remained stable with no clear rise over time. The horizontal line depicts the median. The fold change over control was calculated using the Δ Δ CT method, rats receiving Freund's adjuvant without MPO served as controls. **(B)** Th17 and Th1 cells were present in inflamed kidneys as early as two weeks after immunization with MPO. The fraction of Th1 cells remained on the same level whereas the proportion of Th17 cells sharply increased over time and peaked at week six. T-cells were analyzed by FACS. **(C)** Representative flow cytometric data of renal Th17 cells. The plots are gated on CD45**^+^**CD3**^+^** T-cells. Data is shown as mean with SD- **(D)** MPO-specific renal Th17 and Th1 cells were detectable in MPO-immunized animals but not in controls. Elispot was used to detect antigenspecific T-cells. The horizontal line depicts the median. Mann-Whitney *U* Test was used to calculate statistical significances. A p-value below 0.05 was considered significant. ∗p-value<0.05 ∗∗p-value <0.005.

MPO and control animals were culled at two, four and six weeks after immunization to retrieve the kidneys. Analysis of renal mRNA transcripts showed that IL-17A mRNA transcripts were elevated over controls rising persistently over six weeks (Fig. 2A). In contrast, renal IFNγ mRNA transcripts were stable over time showing on average no increase over controls (Fig. 2A). Interestingly, at four weeks, the renal gene expression for IFNγ and IL-17A was significantly correlated with albuminuria in MPO animals (IFNγ: r = 0.7471, p = 0.0013; IL-17A: r = 0.9265, p < 0.0001). At six weeks, the correlation with IFNγ persisted and was lost for IL-17A (IFNγ: r = 0.5245, p = 0.0085; IL-17A: r = 0.1922, p = 0.3682).At all time-points, T-cells were found in kidneys from MPO and control rats. However, T-cell numbers were very low in kidneys from control rats. The fraction of renal Th17 cells peaked at week six in MPO rats being significantly higher as compared to control rats (% of CD3**^+^** T-cells: 8.87 ± 3.7 % vs. 1.97 ± 0.7 %, p < 0.005, n = 12/11; Fig. 2B). The fraction of renal Th1 cells was stable over time in MPO rats but still significantly higher at weeks four and six as compared to controls (week four, % of CD3**^+^** T-cells: 8.44 ± 1.8 % vs. 2.99 ± 1.2 %, p = 0.009, n = 4/6; week six, % of CD3**^+^** T-cells: 9.75 ± 5.6 % vs. 5.73 ± 4.4 %, p = 0.04, n = 12/11; Fig. 2B). In MPO rats, the proportion of renal Th1 cells was larger than the proportion of Th17 cells only during the first four weeks post immunization. At week six, the average proportion of renal Th1 and Th17 cell was equal in MPO rats (Fig. 2B). MPO-specific renal Th1 and Th17 cells were detectable by EliSpot at weeks four and six post-immunization in MPO-immunized rats being absent in control rats (Fig. 2C).

### 3.3 IL-17A as therapeutic target

**Fig. 3:**
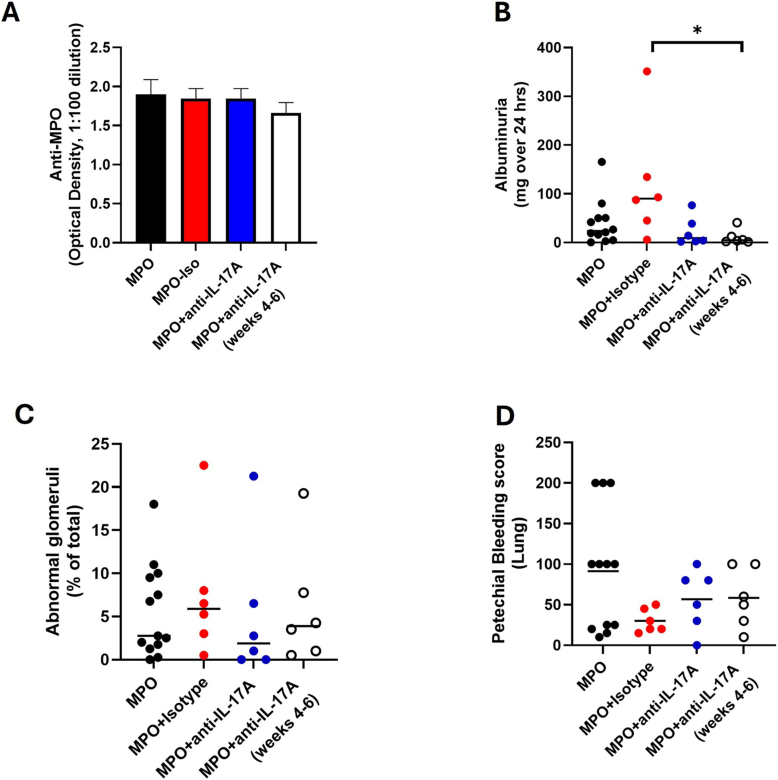
**Neutralization of IL-17A did not affect the development of vasculitis.** Animals were immunized with MPO and received either no treatment (MPO, n = 13), or isotype i.p. injection twice per week (100 μg, n = 6) starting one day before immunization, or anti-IL17A antibody twice per week (100 μg, n = 6) starting one day before immunization or anti-IL17A antibody twice per week (100 μg, n = 6) starting on day 21 after immunization. The horizontal line depicts the median. **(A)** Humoral immunity against MPO was comparable between the treatment groups. Levels were determined by ELISA and samples were diluted 1:100. The optical density is depicted. **(B)** Albuminuria, reflecting renal damage, was higher in MPO-iso as compared to MPO (119.5 ± 121.7 mg/24 h vs. 39.99 ± 46.06 mg/24 h, p = 0.08). Albuminuria was significantly lower in MPO-3W as compared to MPO-iso (11.03 ± 15.1 mg/24 h vs. 119.5 ± 121.7 mg/24 h, p = 0.03) and tended to be lower in MPO-6W as compared to MPO-iso (22.93 ± 29,8 mg/24 h vs. 119.5 ± 121.7 mg/24 h, p = 0.06, Fig. 3B). One-way ANOVA was performed with Tukey's multiple comparisons test for statistical analysis. A p-value below 0.05 was considered significant. **(C)** Renal lesions were not different between the treatment groups. **(D)** The petechial bleeding score was not different between the treatment groups. One-way ANOVA was performed with Tukey's multiple comparisons test for statistical analysis. A p-value below 0.05 was considered significant. ∗p-value<0.05.

**Fig. 4:**
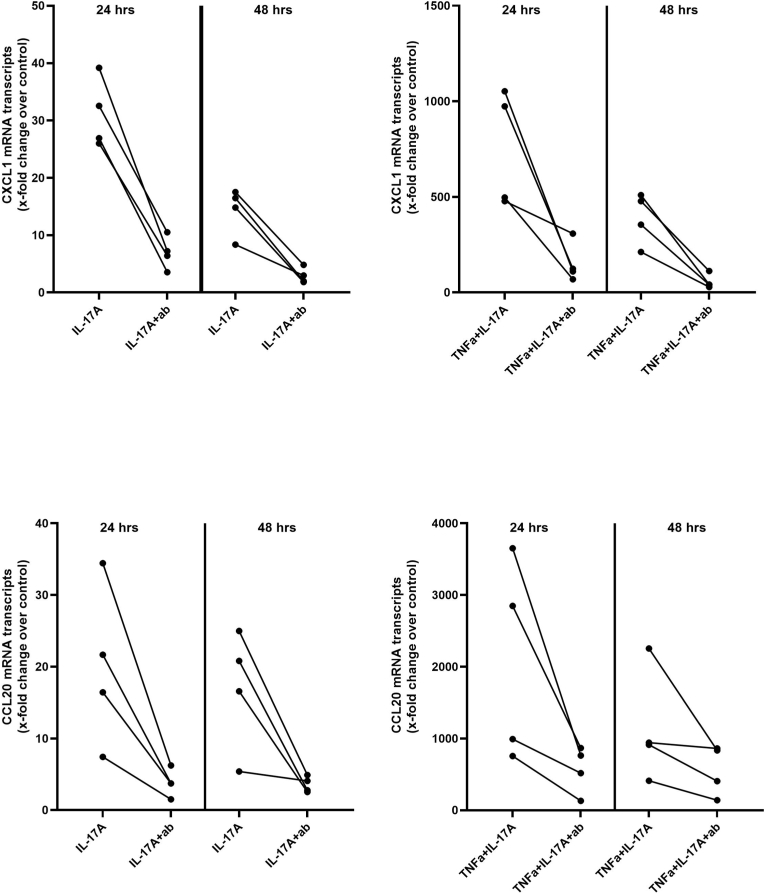
**The anti-IL-17A antibody clone eBio17CK15A5 inhibits the biological activity of IL-*17A in vitro***. **(A,B)** The rat renal tubular cell line (NRK52E cells) was stimulated with IL-17A or TNFα+IL-17A in absence or presence of neutralizing IL-17A antibody (n = 4 independent experiments). Unstimulated cells served as control. The gene expression of the IL-17A target genes CCL20 and CXCL1 served as readout. Stimulation with IL-17A induced the gene expression of CCL20 and CXCL1 already after 24 h. Co-stimulation with TNFα further increased CCL20 and CXCL1 transcripts. The presence of IL-17A antibody led in all cases to a reduction of CCL20 and CXCL1 transcripts confirming the inhibitory activity of the antibody.

As Th17 cells infiltrated the kidney after immunization and were shown to be MPO-specific, it was hypothesized that the signature cytokine IL-17A has a major role in disease pathogenesis. Thus, it was studied whether neutralization of IL-17A has impact on the disease course in this model. For this purpose, MPO animals were either treated with an anti-IL17A antibody for a period of six weeks (MPO-6W, n = 6) starting one day before immunization or received an anti-IL17A antibody for three weeks (MPO-3W, n = 6) starting at day 21 after immunization. The animals were treated with i.p. injections of anti-IL17A twice per week. As control treatment, MPO animals received a matched isotype control (MPO-iso, n = 6) instead of anti-IL17A. In addition, a group immunized with MPO receiving neither isotype nor anti-IL-17A was taken along for control purposes (MPO, n = 13). Independent of anti-IL-17A or control treatment, all animals developed antibodies against MPO. The antibody response did not differ between MPO, MPO-iso and anti-IL17A treatment (Fig. 3A). Petechial bleeding score of the lung and renal histology were not different between MPO-6W/-3W vs. MPO-iso or vs. MPO (Fig. 3C and D). Unexpectedly, albuminuria, reflecting renal damage, was higher in MPO-iso as compared to MPO (119.5 ± 121.7 mg/24 h vs. 39.99 ± 46.06 mg/24 h, p = 0.08, Fig. 3B). Albuminuria was significantly lower in MPO-3W as compared to MPO-iso (11.03 ± 15.1 mg/24 h vs. 119.5 ± 121.7 mg/24 h, p = 0.03) and tended to be lower in MPO-6W as compared to MPO-iso (22.93 ± 29.8 mg/24 h vs. 119.5 ± 121.7 mg/24 h, p = 0.06, Fig. 3B). The antibody used for the *in vivo* experiments inhibited the biological activity of IL-17A *in vitro* (Fig. 4). In rat renal tubular cells (NRK52e cells) stimulated with IL-17A or with a combination of IL-17A and TNFα, the expression of the IL-17A target genes CXCL1 and CCL20 was induced. If cells were stimulated in presence of anti-IL17A antibody, the gene transcripts for CXCL1 and CCL20 were sharply reduced (Fig. 4) confirming the inhibitory activity of this antibody clone.

## 4 Discussion

In this experimental autoimmune vasculitis model, renal vasculitis was accompanied by a strong renal Th17 cell infiltrate. From week two on after immunization with MPO, an enhanced fraction of IL-17A producing intra-renal T-cells was found being highest at week six after immunization. Interestingly, intra-renal Th17 cells were, at least partially, MPO-specific. To study whether the blockade of the signature cytokine IL-17A has impact on disease development and disease course, animals were treated with an anti-IL17A antibody. The antibody was administered either shortly before induction of disease or from week three onward when disease mechanisms were already in effect. Anti-IL17A treatment did not impact development of anti-MPO antibodies, onset of glomerulonephritis or pulmonary vasculitis. However, albuminuria was reduced in the treated animals when compared to animals receiving treatment with isotype.

The animal model used was originally developed by Little et al. and has since been used for mechanistic studies as well as for pre-clinical pilot studies [6,[8], [9], [10]]. Although the disease phenotype is well described with development of renal and pulmonary vasculitis, there is scarce data on the T-cell compartment in this model. Other models of crescentic glomerulonephritis such as the nephrotoxic nephritis model (NTN) or the experimental focal necrotizing GN model have clearly demonstrated that renal inflammation is driven by Th17 cell immunity [11,12]. Mice lacking IL-17A failed to develop crescentic glomerulonephritis while IL-17A competent T-cells transferred from MPO-immunized mice induced renal injury [11,13]. In the rat vasculitis model, this is the first report showing the dynamics of the renal T-cell response. Indeed, it was shown before that circulating Th17 cells are expanded in patients with ANCA-vasculitis and Th17 cells are detectable in the renal infiltrate in ANCA-GN [1,3,5,14,15]. Thus, the model may partly resemble the pathogenesis of ANCA vasculitis. It is a matter of debate whether the renal T-cell response during ANCA-GN is a direct response against the specific autoantigen MPO or PR3. In this EAV model, renal Th17 and Th1 cells were, at least partially, MPO-specific. In principle, MPO-specific T-cells bear the potential to induce proliferative glomerulonephritis in planted-antigen models [16,17]. In the experimental autoimmune glomerulonephritis model, autoantigen-specific T-cells recognizing the alpha-chain of type IV collagen were present within renal lesions [18]. To what extend bystander T-cells have a role in inducing and/or sustaining proliferative glomerulonephritis needs further study.

The dominance of Th17 cells during renal inflammation poses the question whether blockade of the signature cytokine IL-17A has impact on disease mechanisms in the EAV model. Thus, an anti-IL17A antibody was used to treat animals before disease onset and after disease onset. Application of the antibody did not prevent the formation of antibodies against MPO. IL-17A has been implicated in B-cell maturation and plasma cell formation; follicular T-helper cells also express IL-17A [19,20]. Mitsdoerffer et al. showed that transfer of antigen-specific Th17 cells promoted the formation of MOG-specific IgG1 and IgG2b in an autoimmune mouse model [21]. In contrast, humoral immunity against MPO could be induced in Th17 deficient mice as reported by Gan et al. [11]. In the same experimental study, neutralization of IL-12 (to suppress Th1 type immunity) but not IL-23 (to suppress Th17 type immunity) in wild type mice reduced anti-MPO antibody titres [11]. In our studies in the EAV model, induced humoral immunity against MPO may not be entirely dependent of Th17 cells and was not abrogated by IL-17A neutralization. Neutralization of IL-17A did not have significant effects on organ damage in the EAV model. In the anti-MPO GN model by Gan et al., neutralization of IL-23 using anti-IL23p19 antibody treatment ameliorated albuminuria and reduced glomerular lesions [11]. However, a significant treatment effect was only observed during the early phase of anti-MPO GN. Applying the treatment in a later phase rendered IL-23 neutralization ineffective. In our model, albuminuria was highly correlated with renal IL-17A gene expression at week four after immunization but not anymore at week six also suggesting that not the entire pathogenic process is driven by Th17 cells.We chose direct neutralization of IL-17A to suppress Th17 immunity. For that purpose, neutralization was done repetitively twice per week either before induction of disease or after onset of disease beginning on day 21 after immunization. Unexpectedly, treatment did not have impact on the development of humoral anti-MPO; pulmonary and renal vasculitis occurred despite treatment and the timepoint of neutralization did not matter. These findings seem to conflict with previous studies in which IL-17A deficiency conferred protection against the development of murine GN [11,13,18]. However, it needs to be noted that in these studies other animal models were employed that are only partly comparable to the EAV model. These murine models are limited to renal vasculitis and not in all animal models MPO-specific immunity is induced. Thus, this methodological difference may explain the divergent findings. In addition, targeted therapy with an anti-cytokine antibody is not the same as genetic deficiency. Anti-cytokine treatment may not abrogate binding of the target molecule to its receptor to suppress the cascade mediated by the targeted cytokine. Cytokine/antibody complexes may even enhance cytokine/receptor signalling [22]. Therefore, neutralizing activity *in vitro* does not necessarily imply neutralization efficacy *in vivo*. Furthermore, redundant effector mechanisms of vasculitic inflammation may reduce the efficacy of simple cytokine neutralization. Accordingly, research work by Kitching and colleagues demonstrated the importance of Th1 responses for renal vasculitis in addition to Th17 [23,24]. In a recent case series, based on a sophisticated profiling approach, an antibody targeting IL-23 (Th17) and IL-12 (Th1) at the same time, was used for induction therapy in combination with low dose cyclophosphamide to treat four ANCA patients [15]. In all patients, clinical improvement was achieved suggesting that a combined blockade of Th17 and Th1 immunity may be efficacious.

Furthermore, animals undergoing anti-IL-17A treatment had lower albuminuria than animals receiving control, i.e. isotype, treatment after immunization with MPO. However, these isotype treated animals showed higher albuminuria than MPO-immunized animals not receiving any kind of antibody treatment. Thus, the meaning of the reduction in albuminuria in anti-IL17A treated remains unclear; the observation that neither anti-10.13039/501100004578MPO levels nor histological lesions were reduced in the same fashion does not support a relevant treatment effect in this time frame. Nevertheless, it might be worthwhile to investigate the effects on a longer time scale as reduction in albuminuria may precede treatment effects on histological and immunological level. It needs to be emphasized that the animal model used, as it is the case with all other animal models, only partly resembles the pathogenesis of ANCA-models and a direct translation of the findings to the human situation is not possible.

In summary, the dynamics of T-cell immunity in the EAV model shows a major participation of MPO-specific Th17 and Th1 cells in renal vasculitis. Simple cytokine neutralization was not efficacious in this disease model so that combined neutralization approaches should be studied further in this model.

## CRediT authorship contribution statement

**Ye Zeng:** Writing – review & editing, Writing – original draft, Methodology, Investigation, Formal analysis, Data curation, Conceptualization. **Erika Boschmann:** Writing – review & editing, Writing – original draft, Investigation, Formal analysis, Data curation. **Julia Kotte:** Writing – review & editing, Writing – original draft, Investigation, Formal analysis, Data curation. **Ming Sun:** Writing – review & editing, Writing – original draft, Investigation, Formal analysis, Data curation. **Lennart Hess:** Writing – review & editing, Writing – original draft. **Sebastian Dolff:** Writing – review & editing, Writing – original draft, Methodology, Investigation, Conceptualization. **Tanja Hinkeldein:** Writing – review & editing, Writing – original draft, Project administration, Methodology, Investigation, Formal analysis, Data curation. **Janna Hagedorn:** Writing – review & editing, Investigation. **Robert Langer:** Writing – review & editing, Writing – original draft, Methodology, Investigation, Formal analysis, Conceptualization. **Pieter van Paassen:** Writing – review & editing, Writing – original draft, Methodology, Conceptualization. **Jan Damoiseaux:** Writing – review & editing, Writing – original draft, Methodology. **Jan Willem Cohen Tervaert:** Writing – review & editing, Writing – original draft, Methodology, Investigation. **Oliver Witzke:** Writing – review & editing, Writing – original draft, Resources, Conceptualization. **Andreas Kribben:** Writing – review & editing, Writing – original draft, Resources, Conceptualization. **Benjamin Wilde:** Writing – review & editing, Writing – original draft, Supervision, Methodology, Investigation, Funding acquisition, Formal analysis, Data curation, Conceptualization.

## Declaration of competing interest

The authors declare the following financial interests/personal relationships which may be considered as potential competing interests: Benjamin Wilde reports financial support was provided by Doctor Werner Jackstadt Trust. Oliver Witzke reports financial support was provided by 10.13039/501100014837Rudolf Ackermann Foundation. If there are other authors, they declare that they have no known competing financial interests or personal relationships that could have appeared to influence the work reported in this paper.

## Data Availability

Data will be made available on request.
